# Natural quantitative genetic variance in plant growth differs in response to ecologically relevant temperature heterogeneity

**DOI:** 10.1002/ece3.2482

**Published:** 2016-10-01

**Authors:** Matti J. Salmela, Brent E. Ewers, Cynthia Weinig

**Affiliations:** ^1^ Department of Botany University of Wyoming Laramie WY USA; ^2^ Program in Ecology University of Wyoming Laramie WY USA; ^3^ Department of Molecular Biology University of Wyoming Laramie WY USA; ^4^ Present address: Natural Resources Institute Finland Vantaa Finland

**Keywords:** environmental heterogeneity, genotype × environment interaction, maintenance of genetic diversity, temperature, temporal heterogeneity

## Abstract

Adaptation to large‐scale spatial heterogeneity in the environment accounts for a major proportion of genetic diversity within species. Theory predicts the erosion of adaptive genetic variation on a within‐population level, but considerable genetic diversity is often found locally. Genetic diversity could be expected to be maintained within populations in temporally or spatially variable conditions if genotypic rank orders vary across contrasting microenvironmental settings. Taking advantage of fine‐resolution environmental data, we tested the hypothesis that temperature heterogeneity among years could be one factor maintaining quantitative genetic diversity within a natural and genetically diverse plant population. We sampled maternal families of *Boechera stricta*, an *Arabidopsis thaliana* relative, at one location in the central Rocky Mountains and grew them in three treatments that, based on records from an adjacent weather station, simulated hourly temperature changes at the native site during three summers with differing mean temperatures. Treatment had a significant effect on all traits, with 2–3‐fold increase in above‐ and belowground biomass and the highest allocation to roots observed in the treatment simulating the warmest summer on record at the site. Treatment affected bivariate associations between traits, with the weakest correlation between above‐ and belowground biomass in the warmest treatment. The magnitude of quantitative genetic variation for all traits differed across treatments: Genetic variance of biomass was 0 in the warmest treatment, while highly significant diversity was found in average conditions, resulting in broad‐sense heritability of 0.31. Significant genotype × environment interactions across all treatments were found only in root‐to‐shoot ratio. Therefore, temperature variation among summers appears unlikely to account for the observed levels of local genetic variation in size in this perennial species, but may influence family rank order in growth allocation. Our results indicate that natural environmental fluctuations can have a large impact on the magnitude of within‐population quantitative genetic variance.

## 1 Introduction

In the wild, native populations often exhibit a fitness advantage in their home environments. This phenomenon, local adaptation, is frequently documented among populations found across heterogeneous environments and is known to maintain genetic diversity in fitness and many other quantitative traits among populations (Hereford, 2009; Leimu & Fischer, 2008). On a more local within‐population level, however, natural selection is expected to reduce genetic variation as nonoptimal phenotypes are removed from populations (Kawecki & Ebert, 2004). In contrast to these predictions, substantial quantitative genetic variation is frequently found within populations, even in traits closely related to fitness that are assumed to experience strong selection (Barton & Keightley, 2002; Hill, 2010; Houle, 1992; Johnson & Barton, 2005). For example, flowering time exhibits genetic diversity not just among but also within populations in the mainly self‐fertilizing model species *Arabidopsis thaliana* (L.) Heynh. in Fennoscandia and Spain (Kuittinen, Mattila, & Savolainen, 1997; Méndez‐Vigo, Gomaa, Alonso‐Blanco, & Pico, 2013; Stenøien, Fenster, Tonteri, & Savolainen, 2005), while variation in timing of growth is often distributed in a similar fashion in highly outcrossing forest trees (Alberto et al., 2011; Mimura & Aitken, 2007; Savolainen, Bokma, García‐Gil, Komulainen, & Repo, 2004).

In spite of extensive theoretical work on the potential of different evolutionary and ecological forces to maintain genetic variation within populations, the causes of such local diversity remain for the most part poorly understood (Johnson & Barton, 2005). Mutation‐selection balance on its own is considered unlikely to account for the observed high levels of genetic diversity, while various forms of balancing selection have been hypothesized to play a causal role (Barton & Keightley, 2002; Charlesworth, 2015; Hill, 2010; Johnson & Barton, 2005). Further, gene flow among genetically distinct populations from divergent environments may be a source of variation within populations (Barton, 1999). Because a population's evolutionary potential in a changing environment is determined largely by the magnitude of heritable genetic diversity in adaptive traits (Falconer & Mackay, 1996), the maintenance of genetic variation is relevant not only to basic research on evolutionary processes but also to applied conservation biology and rare species’ management (Hoffmann & Sgrò, 2011). Indeed, empirical evidence that at least some populations in the wild have been able to adapt to environmental changes indicates that adaptive genetic diversity existed in previous generations (Franks, Weber, & Aitken, 2014; Merilä & Hendry, 2014; see also Geerts et al., 2015; Irwin, Finkel, Müller‐Karger, & Ghinaglia, 2015).

In addition to large‐scale spatial variation in the environment that often leads to genetically differentiated populations, environmental conditions may also vary at much finer spatial and temporal scales (Linhart & Grant, 1996). Consequently, a population may encounter variable conditions at its home site, which could select for the potential to express a wider range of phenotypes across distinct microenvironments. Such phenotypic variation could be achieved for instance by genetic diversity such that different genotypes have the highest fitness in differing local environmental settings, that is, by genotype × environment interactions (Ellner & Hairston, 1994; Gillespie & Turelli, 1989; Hedrick, 1995), or via phenotypic plasticity of a generalist genotype (Kawecki & Ebert, 2004). Empirical field studies have shown that performance ranks of genotypes may change as a result of variation in inter‐ and intraspecific competition (Baron, Richirt, Villoutreix, Amsellem, & Roux, 2015; Shaw, Platenkamp, Shaw, & Podolsky, 1995) or disturbance (McLeod, Scascitelli, & Vellend, 2012); genotype × environment interactions in fitness have even been described on a scale of just 10 cm within a single old field (Stratton, 1994). In *Betula pendula* Roth in Finland, however, forest ground heterogeneity on a local level affected overall growth but was not sufficient to shift genotypic ranks (Mikola et al., 2014). Genetic mapping studies on recombinant inbred lines and experimental hybrids of model systems such as *Arabidopsis, Boechera*,* Drosophila*, and *Mimulus* in the field and under controlled experimental conditions have provided insights into the genetic basis of genotype × environment interactions across highly divergent environmental conditions. These studies commonly show environment‐specific QTL effects for numerous quantitative traits from morphology to reproductive fitness (reviewed in Mackay, 2009; Savolainen, Lascoux, & Merilä, 2013; Weinig, Ewers, & Welch, 2014).

Genetically variable populations of predominantly self‐fertilizing plant species like the mustard *Boechera stricta* (Graham) Al‐Shehbaz (Song, Clauss, Pepper, & Mitchell‐Olds, 2006) are suitable study systems for exploring the maintenance of local genetic diversity in varying environments because long‐distance gene flow may be reasonably excluded as the source of high levels of within‐population adaptive genetic diversity (cf. Yeaman & Jarvis, 2006). Previously, Salmela et al. (2016) observed considerable quantitative genetic diversity in the circadian clock, the endogenous timekeeper regulating daily oscillations in numerous traits, and various growth traits among maternal families of *B. stricta* sampled at a high‐elevation site in the central Rocky Mountains in the USA. In this population, the range of family means in circadian period, one characteristic of the circadian clock, accounted for over 50% of the range that has previously been reported in a global set of 150 *A. thaliana* genotypes by Michael et al. (2003). Quantitative variation in the clock was associated with growth so that families with longer circadian periods grew more rapidly and to a larger size, but had a lower root‐to‐shoot ratio. Because the circadian clock and its variability are generally thought to reflect adaptation to variable light–dark cycles (e.g., Michael et al., 2003), Salmela et al. (2016) hypothesized that local genetic diversity in the clock could be maintained by performance trade‐offs of families across seasonal environments with differing photoperiods and temperatures. Yet, growth chamber environments simulating different months within a growing season resulted in genotype × environment interactions that were larger than the overall genetic effect only for root‐to‐shoot allocation. Higher root‐to‐shoot ratio may enhance survival in stressful and low‐resource environments (Poorter et al., 2012; Wilson, 1988), an adaptive hypothesis also suggested by the larger root‐to‐shoot ratios found in populations from higher‐elevation environments in this region. Still, the exact factors enabling the maintenance of significant fine‐scale genetic diversity in this population remain unknown.

Fluctuating selection driven by temporal variation in environmental factors has been proposed to be common in the wild (Siepielski, DiBattista, & Carlson, 2009). Temporally variable environmental conditions have also been hypothesized to shape the patterns of adaptive genetic diversity within populations (Salmela, 2014), but in general, theoretical studies suggest that conditions under which temporal variation in the environment can maintain genetic diversity are more restrictive than those for spatial heterogeneity (Hedrick, 2006). Some evidence supports the role of temporal environmental heterogeneity in maintaining genetic variation within populations: Different growth seasons within a year have been reported to favor divergent genotypes in *Taraxacum officinale* (L.) Weber ex F.H. Wigg (Vavrek, McGraw, & Yang, 1996), while annually varying moisture conditions in a desert may conserve flower color polymorphism in the annual *Linanthus parryae* (A.Gray) Greene (Schemske & Bierzychudek, 2001). In the fish *Xiphophorus variatus*, the level of temperature variability within days and years has been found to be positively associated with within‐population diversity in tail spot richness (Culumber & Tobler, 2016), with the fitness rank of different spot types depending for instance on the thermal environment (Culumber, Schumer, Monks, & Tobler, 2015).

In this study, we were interested in further exploring the capacity of temporal abiotic variability to sustain local quantitative genetic variation in the wild using the aforementioned *B. stricta* population from the central Rocky Mountains as our study system. Two features in particular make this population an interesting model for assessing the possible environmental causes of local genetic diversity: It has already been shown to be genetically diverse in multiple quantitative traits (Salmela et al., 2016), and its home site is located close to a weather station with long‐term and fine‐resolution temperature records. The population experiences for instance variable summer temperature conditions; between 1995 and 2014, mean June and July temperatures at the site varied between 8.3–15°C and 13–17.2°C, respectively. Hourly recorded temperature data from multiple years allowed us to investigate the effects of heterogeneity in a single abiotic factor, providing the unique opportunity to simulate temporally fluctuating and ecologically relevant natural environments in controlled experimental settings. We grew replicates of the same set of naturally occurring maternal families across treatments with differing average temperatures, hypothesizing that if variable summer temperature regimes were capable of preserving quantitative genetic diversity in this population, not only would the treatments affect overall means but also induce genotype × environment interactions in growth and its allocation. Moreover, we expected the magnitude of these interactions to be large compared to the average effect of family and a large proportion of the interaction variance to be due to rank shifts of families among simulated growing season temperature cycles.

## 2 Materials and methods

### 2.1 Sampling

We collected seed material for the study by maternal family at a distance of about 300–500 m from the South Brush Creek SNOTEL weather station (41.333°N, 106.500°W; elevation 2,572 m) in southeastern Wyoming on 26 July 2012. The inbreeding coefficient in populations of *B. stricta* is high (0.74–0.98 in Song et al., 2006), suggesting that maternal progeny consist mostly of full‐sibs. All except one of the sampled plants were located along a transect of approximately 300 m. Because we were interested in natural patterns of variation present in the population at the time of sampling rather than characterizing the genetic background of quantitative trait variation, we used wild‐collected seeds in the experiment. Of the 21 families investigated in this study, 19 were included in the study by Salmela et al. (2016) that documented significant within‐population genetic variation in the circadian clock and growth.

### 2.2 Treatments

**Figure 1:**
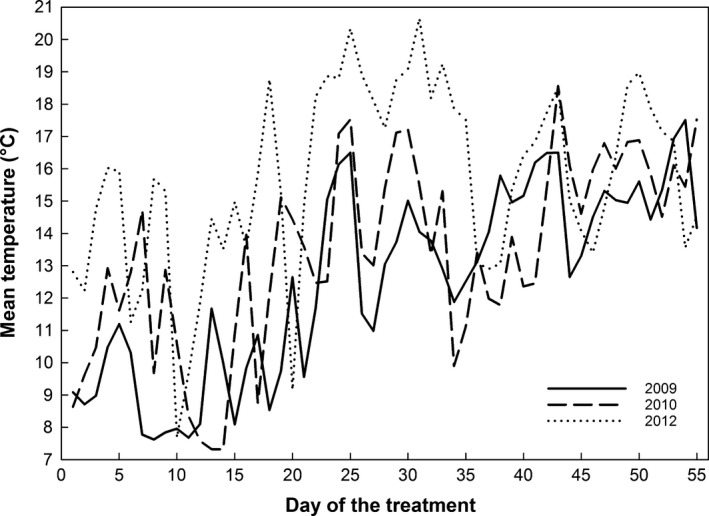
Variation in daily mean temperatures in growth chamber treatments simulating three summers with differing average temperatures at South Brush Creek in southeastern Wyoming. Overall treatment means were 12.5°C in 2009, 13.4°C in 2010, and 15.7°C in 2012. Due to the hourly tracking of temperature heterogeneity at the site, each treatment consisted of over 1,300 different temperature steps

Based on records from the weather station (http://www.wcc.nrcs.usda.gov/nwcc/site?sitenum=772), we programmed growth chamber compartments (PGC‐9/2 with Percival Advanced Intellus Environmental Controller, Percival Scientific, Perry, IN, USA) to track hourly temperature changes at the site during an 8‐week period on June 1–July 25 in 2009, 2010, and 2012. Thus, each treatment consisted of over 1,300 temperature steps. In the following sections of the article, we will refer to these treatments by the corresponding year. We chose to focus on June and July because an earlier study indicated the most vigorous growth in a treatment simulating early growing season conditions (Salmela et al., 2016) and because in May the site regularly experiences freezing temperatures as low as −15°C that cannot be achieved in the chambers used. Daily mean temperatures in each treatment are shown in Figure 1. These treatments represented a range of mean June temperatures at the site (10.2°C in 2009, 12.0°C in 2010, and 15.0°C in 2012) during a period from which hourly recorded temperature data were available; the summer of 2012 is the hottest on record at the site and in the rest of Wyoming (NOAA National Overview for Annual 2012). Due to lack of hourly data, we did not simulate the coolest June on record (1998, with average temperature of 8.3°C). Average daily maximum and minimum temperatures in June were 17.4°C and 3.6°C in 2009, 19.6°C and 4.2°C in 2010, and 23.4°C and 5.1°C in 2012. Under chamber conditions, mean July temperatures were 14.7°C in both 2009 and 2010, and 16.3°C in 2012. This resulted in overall treatment means of approximately 12.5°C in 2009, 13.4°C in 2010, and 15.7°C in 2012. Due to chamber restrictions on minimum temperature (4°C in the dark, 10°C with all lights on), overall experimental conditions were slightly (0.09–0.23°C) higher than those in the field.

Photoperiod was approximately 15 hr, with gradual changes in a similar fashion in all treatments according to local sunrise and sunset times over the course of the 8‐week period. To simulate dawn and dusk, 50% of the lights were on during the first and last hour of the photoperiod. Maximum photosynthetically active irradiance, measured with the light meter LI‐250 (LI‐COR Biosciences, Lincoln, NE, USA) in all chambers before the experiment, was approximately 250 μmol photons m^−2^ s^−1^ at the plant level. We did not replicate treatments; thus, family × treatment effects cannot be unequivocally attributed to temperature. However, variation in other controllable factors is likely to have been small compared to temperature due to very similar light levels in all chamber compartments. Daily monitoring of temperature by thermometers also indicated that chamber conditions adhered to programmed settings.

We germinated and planted seeds from the 21 families, with ten replicates per family in each treatment, following the protocol in Salmela et al. (2016). We did not score timing of germination due to rapid germination on moist paper in all the families within 4 days following cold stratification. We divided the seedlings into the three treatments, with ten blocks within each and one replicate per family randomized within a block. We watered pots to field capacity every 2 days and rotated flats within the chamber compartments twice every week. The species requires vernalization in order to flower; due to difficulties in simulating ecologically relevant high‐elevation autumn, winter, and spring conditions in our experimental settings (air temperature at the site can drop below −30°C), we measured the equivalent of first‐year growth only. After 8 weeks, we scored the number of leaves and the length of the longest leaf in all replicates and sampled whole plants for above‐ and belowground biomass and root‐to‐shoot ratio. We dried plants in an oven at 65°C for 3 days before biomass measurements. Leaf measures were strongly correlated with aboveground biomass (the number of leaves: *r* ≥ .807, *p* < .0001 in all treatments; the longest leaf length *vs*. √aboveground biomass: *r* ≥ .923, *p* < .0001 in all treatments). Because qualitatively similar results were obtained with all correlated measures, we will use biomass only as a measure of aboveground plant growth. We sampled plants at 8 weeks because the oldest leaves often start to senesce at this point in chamber conditions (M.J. Salmela, personal observation) and because an 8‐week period is likely to account for a large proportion of the annual growing season at the home site of the population.

### 2.3 Statistical analyses

We included families with at least four replicates in two treatments in the analysis. Prior to formal statistical tests, we examined whether our data met assumptions of analysis of variance. We detected variance heterogeneity of residuals among treatments in all traits, with variances varying 6.6‐fold for aboveground biomass, 9.5‐fold for belowground biomass, and 4.8‐fold for root‐to‐shoot ratio. These ranges of variation resulted from the 2012 treatment that had the most phenotypic variation in all traits; variance differences between 2009 and 2010 were less than twofold. To determine the influence of unequal variances, we ran all statistical analyses with the complete dataset using both original and transformed measurements. While transformations reduced variance heterogeneity, they did not change the outcome of the analyses or interpretation of the results. This outcome probably reflects the robustness of analysis of variance to unequal variances in the case of similar sample sizes in all treatments. Therefore, we used the raw values in the analyses presented here.

We first tested for the significant effects of family and block within each treatment using general linear models. We consider variation among maternal families to represent genetic diversity within the population, and thus, proportion of total variation explained by among‐family diversity in each treatment is an estimate of broad‐sense heritability (H^2^). We estimated variance components for family, block, and residual variation using the REML approach. We examined associations between the traits within treatments using replicate values and Pearson's correlation. Scatterplots suggested the slope between above‐ and belowground biomass varies among treatments. To test for treatment differences in slope (ß_1_), we performed a regression analysis of aboveground biomass on belowground biomass in which we included treatment as a categorical factor and the aboveground biomass × treatment interaction term.

To investigate the contributions of different sources of variation across all three treatments and between pairs of treatments, we used the model above‐ or belowground biomass or root‐to‐shoot ratio = treatment + family + family × treatment + block(treatment). We considered treatment a fixed factor. When nonsignificant at the 0.10 level, we removed the interaction term from the analysis. Given that among‐family variation measures genetic diversity within the population, the family × treatment term defines genotype × environment interactions. We estimated variance components for the random factors using the REML approach. Genotype × environment interactions can maintain diversity if they are caused by rank shifts of genotypes rather than by differences in among‐genotype variance among treatments (Mitchell‐Olds, 1992). In order to separate the effects of shifts in family rank order *vs*. among‐family variance on a statistically significant interaction variance component, we used the equation (Cockerham, 1963):

$$V_{\mathrm{Family}\;\times \;\mathrm{treatment}}=\left(\sum_{i<j}\;\left[2\sigma_{i}\sigma_{j}\;\left(1-r_{\mathrm{GE}}\right)+{\left(\sigma_{i}-\sigma_{j}\right)}^{2}\right]\right)/(t\left(t-1\right),$$

where *V*_Family × treatment_ is the variance component due to family × treatment interaction, σ_*i*_ and σ_*j*_ are the square roots of among‐family variances in treatments *i* and *j,* r_GE_ is the genetic correlation between treatments *i* and *j*, and *t* is the number of treatments. The first part of the equation accounts for variance due to changes in rank order among treatments, while the second part accounts for treatment differences in among‐family variance. We estimated genetic correlations using family variance components within each treatment and across pairs of treatments (Windig, 1997):

$$r_{\mathrm{GE}}=V_{\mathrm{Family}}/\sqrt{(V_{\mathrm{Family},\;\mathrm{treatment}\;i}\times V_{\mathrm{Family},\;\mathrm{treatment}\;j})},$$

where V_Family_ is the variance component due to family across the two environments, and *V*_Family, treatment *i*_ and *V*_Family, treatment *j*_ are the variance components due to family in treatments *i* and *j*. We carried out all statistical analyses with IBM SPSS Statistics Version 23.

## 3 Results

Table 1 shows general linear model results for the three traits within each treatment, along with treatment means and measures of phenotypic and genetic diversity. In 2009, the family effect was significant for aboveground biomass and root‐to‐shoot ratio, an indication of genetic variation in the population. For belowground biomass, the family effect was close to significance at the 0.05 level (*p* = .078). In 2010, the family effect was highly significant for all traits. Although 2012 was the most phenotypically variable treatment, we found no significant genetic variation for above‐ or belowground biomass in this year; variation was entirely residual. For root‐to‐shoot ratio, however, family differences in 2012 were highly significant. For aboveground biomass, phenotypic and genetic variation in 2009 were 56.3% and 18.0%, respectively, of those in 2010, resulting in higher broad‐sense heritability in 2010. The pattern was similar for belowground biomass: Phenotypic and genetic variation in 2009 were 64.0% and 16.0%, respectively, of those in 2010. These differences were not due to the two families in 2010 for which we lacked data in 2009; when they were excluded, we obtained very similar estimates of within‐treatment diversity. Similar levels of genetic diversity were expressed in root‐to‐shoot ratio in 2009 and 2010, but broad‐sense heritability in 2009 was lower due to 66.5% more phenotypic variation in this treatment. We observed the highest phenotypic and genetic variation in root‐to‐shoot ratio in 2012. However, due to increases of similar magnitude in all variance components, broad‐sense heritability in 2012 was similar to that in 2010.

**Table 1:** General linear model results for above‐ and belowground biomass and root‐to‐shoot ratio in a population of *Boechera stricta* within each treatment simulating differing growing season temperature conditions at South Brush Creek in southeastern Wyoming

Factor	df	Aboveground biomass				Belowground biomass				Root‐to‐shoot ratio			
		MS	*F*‐ratio	*p*‐value	%	MS	*F*‐ratio	*p*‐value	%	MS	*F*‐ratio	*p*‐value	%
2009													
Family	18	0.00124	1.84	*	9.96^a^	0.0000557	1.58	.0784	6.53^a^	0.00734	2.18	**	11.7^a^
Block	9	0.00119	1.76	.0840	4.92	0.0000607	1.72	.0931	4.89	0.0111	3.30	**	11.9
Residual	105	0.000674			85.1	0.0000352			88.6	0.00336			76.3
Mean (±*SE*)		0.0557 (±0.00243) g				0.0111 (±0.000547) g				0.202 (±0.00570)			
*V*_P_		7.99 × 10^−4^				4.02 × 10^−5^				4.43 × 10^−3^			
*V*_F_		7.96 × 10^−5^				2.63 × 10^−6^				5.21 × 10^−4^			
2010													
Family	20	0.00460	5.35	****	31.0^a^	0.000179	4.52	****	26.2^a^	0.00590	3.24	****	20.1^a^
Block	9	0.00311	3.61	***	8.84	0.000163	4.10	***	11.0	0.00659	3.62	***	11.4
Residual	147	0.000860			60.2	0.0000396			62.8	0.00182			68.5
Mean (±*SE*)		0.0573 (±0.00282) g				0.0105 (±0.000593) g				0.174 (±0.00384)			
*V*_P_		1.42 × 10^−3^				6.28 × 10^−5^				2.66 × 10^−3^			
*V*_F_		4.41 × 10^−4^				1.64 × 10^−5^				5.35 × 10^−4^			
2012													
Family	19	0.00469	0.947	ns	0^a^	0.000377	0.957	ns	0^a^	0.0275	3.04	***	19.5^a^
Block	9	0.000855	0.173	ns	0	0.000200	0.508	ns	0	0.0298	3.29	**	11.2
Residual	120	0.00495			100	0.000394			100	0.00906			69.3
Mean (±*SE*)		0.115 (±0.00561) g				0.0311 (±0.00160) g				0.268 (±0.00934)			
*V*_P_		4.69 × 10^−3^				3.83 × 10^−4^				1.30 × 10^−2^			
*V*_F_		0				0				2.52 × 10^−3^			

The percentage shows the proportion of total variation explained by each factor. *V*_P_ = variance component for phenotypic variation; *V*_F_ = variance component for among‐family (genetic) variation.

ns = *p* > .10, *p < .05, **p < .01, ***p < .001, ****p < .0001.

^a^Estimate of broad‐sense heritability (H^2^).

**Figure 2:**
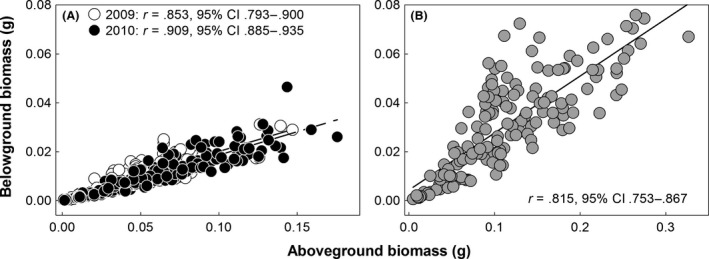
Variable associations between above‐ and belowground biomass within a population of *Boechera stricta* sampled at South Brush Creek in southeastern Wyoming and grown in three treatments simulating differing summer temperature conditions at its native site. 2012 differed from the two other treatments in its overall trait means and variances. For clarity, we show data for 2012 in a separate figure. (A) In treatments simulating the summers of 2009 and 2010, the correlations and slopes between the traits were not significantly different from each other. (B) In the treatment simulating the summer of 2012, the correlation between the traits was significantly weaker than in 2010. Also, the slope was significantly steeper (ß_1_ = 0.233) than the one in 2010 (ß_1_ = 0.191)

Above‐ and belowground biomass were positively correlated in all treatments (Figure 2). This association was the strongest in 2010 (Figure 2A) and the weakest in 2012 (Figure 2B). A significant aboveground biomass × treatment interaction in the regression analysis indicated treatment differences in the slope between the two variables (*F*_2,453_ = 4.13, *p* < .05), with very similar estimates in 2009 and 2010 (2009: ß_1_ = 0.192, 95% CI: 0.171–0.212; 2010: ß_1_ = 0.191, 95% CI: 0.178–0.204; Figure 2A) and a steeper one in 2012 (ß_1_ = 0.233, 95% CI: 0.206–0.260; Figure 2B). Belowground biomass was positively and moderately correlated with root‐to‐shoot ratio in all treatments (2009: *r* = .470, *p* < .0001, 95% CI: 0.330–0.605; 2010: *r* = .592, *p* < .0001, 95% CI: 0.482–0.689; 2012: *r* = .569, *p* < .0001, 95% CI: 0.464–0.664). In 2010, aboveground biomass showed a weak positive correlation with root‐to‐shoot ratio (*r* = .288, *p* < .001, 95% CI: 0.153–0.422). In 2009 and 2012, this correlation was not significantly different from zero.

**Figure 3:**
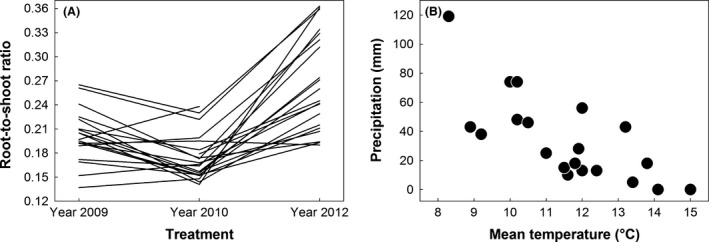
(A) Reaction norms of root‐to‐shoot ratio for naturally occurring maternal families of *Boechera stricta* that were sampled at South Brush Creek in southeastern Wyoming and grown in three treatments simulating summers with differing average temperatures at the population's home site. (B) The negative association between mean June temperature and precipitation during the month at South Brush Creek between 1995 and 2014 (*r* = −.737, *p* < .001)

Treatment had a highly significant effect on all three traits (Table 2). Aboveground biomass was twice and belowground biomass about three times larger in 2012 than in 2009 or 2010, and thus, also root‐to‐shoot ratio displayed its highest treatment mean in 2012 (Table 1). For above‐ and belowground biomass, the only significant random factor across all treatments was family. We found a significant family × treatment interaction term for root‐to‐shoot ratio, revealing variation in phenotypic plasticity among maternal families in the population. The variance component due to the interaction was about 27% bigger (6.49 × 10^−4^) than that of family (5.10 × 10^−4^). Approximately 63% of the interaction variance was due to rank changes of families across the treatments (Figure 3A), resulting in very similar magnitude of variance components due to family *vs*. rank order changes. In accordance with the moderate overall family effect, genetic correlations for root‐to‐shoot ratio between treatments were positive: 0.720 between 2009 and 2010, 0.527 between 2009 and 2012, and 0.521 between 2010 and 2012.

**Table 2:** General linear model results for above‐ and belowground biomass and root‐to‐shoot ratio across all treatments

Factor	df	Aboveground biomass			Belowground biomass			df	Root‐to‐shoot ratio		
		MS	*F*‐ratio	*p*‐value	MS	*F*‐ratio	*p*‐value		MS	*F*‐ratio	*p*‐value
Treatment	2	0.156	87.1	****	0.0193	128	****	2	0.344	17.7	****
Family	20	0.00582	2.71	***	0.000256	1.65	*	20	0.0215	2.29	*
Family × treatment								37	0.00950	2.07	***
Block(treatment)	27	0.00177	0.822	ns	0.000151	0.972	ns	27	0.0158	3.45	****
Residual	409	0.00215			0.000155			372	0.00459		

ns = *p* > .10, * *p* < .05, ** *p* < .01, *** *p* < .001, **** *p* < .0001.

Interaction terms were included in the models when significant at the *p* < .10 level.

Table 3 shows results for pairwise comparisons of treatments. We found highly significant treatment differences in biomass between 2009 and 2012, and 2010 and 2012. Although the overall effect of treatment was not significant in the comparison of 2009 and 2010, we observed a significant family × treatment interaction for aboveground biomass. However, the variance component due to family was 1.73 times bigger than that due to the interaction, signaling that the significant interaction arises mainly from the treatment differences in among‐family variance (Table 1). For belowground biomass in the same treatment comparison, the pattern was comparable: The family × treatment interaction was close to significance at the 0.05 level (*p* = .0880). For root‐to‐shoot ratio, pairwise comparisons of treatments indicated moderate differences in mean between 2009 and 2010, and highly significant differences between 2009 and 2012, and 2010 and 2012. The difference between 2009 and 2010, despite the lack of significant treatment effects in its two components, was likely caused by small but opposite changes in the two biomass measures: Overall aboveground biomass was slightly larger in 2010 than in 2009, while the opposite pattern was found for belowground biomass (Table 1). In the comparison of 2009 and 2010 to 2012, we detected some support for a family × treatment interaction (*p* = .0941). In the pairwise comparisons of 2009 and 2010 to 2012, we observed more statistical support for family × treatment interactions than for family. Together with variation in the strength of genetic correlations, the results indicate that differing family responses to 2012 in particular account for the significant interaction term in root‐to‐shoot ratio across all treatments.

**Table 3:** General linear model results for pairwise treatment comparisons

Factor	2009–2010				2009–2012				2010–2012			
	df	MS	*F*‐ratio	*p*‐value	df	MS	*F*‐ratio	*p*‐value	df	MS	*F*‐ratio	*p*‐value
Aboveground biomass												
Treatment	1	0.000417	0.159	ns	1	0.208	164	****	1	0.244	121	****
Family	20	0.00382	2.62	*	20	0.00386	1.34	ns	20	0.00582	2.12	**
Family × treatment	18	0.00146	1.86	*								
Block(treatment)	18	0.00215	2.74	***	18	0.00105	0.362	ns	18	0.00196	0.715	ns
Residual	252	0.000783			242	0.00289			286	0.00275		
Belowground biomass												
Treatment	1	0.0000261	0.217	ns	1	0.0244	165	****	1	0.0317	166	****
Family	20	0.000158	2.77	*	20	0.000224	0.992	ns	20	0.000287	1.42	ns
Family × treatment	18	0.0000569	1.51	.0880								
Block(treatment)	18	0.000112	2.95	****	18	0.000137	0.608	ns	18	0.000190	0.935	ns
Residual	252	0.0000378			242	0.000226			286	0.000203		
Root‐to‐shoot ratio												
Treatment	1	0.0488	5.31	*	1	0.287	11.5	**	1	0.687	29.3	****
Family	20	0.00935	2.55	*	20	0.0213	1.62	ns	20	0.0212	1.83	.0949
Family × treatment	18	0.00367	1.49	.0941	17	0.0131	2.05	**	19	0.0117	2.30	**
Block(treatment)	18	0.00883	3.59	****	18	0.0204	3.19	****	18	0.0182	3.59	****
Residual	252	0.00246			225	0.00640			267	0.00507		

ns = *p* > .10, *p < .05, **p < .01, ***p < .001, ****p < .0001.

Interaction terms were included in the models when significant at the *p* < .10 level.

## 4 Discussion

In this study, we combined environmental data with quantitative genetic approaches to examine whether temporal environmental heterogeneity could act as one mechanism maintaining genetic variation in growth and its allocation within a plant population experiencing variable conditions at its home site. In order to carry out the experiment in as ecologically relevant chamber conditions as possible, we took advantage of fine‐resolution temperature data from a weather station that was located in the vicinity of the sampled population. This enabled us to quantify the potential of natural among‐year heterogeneity in growing season temperatures to induce genotype × environment interactions in first‐year growth traits.

Self‐fertilization is expected to reduce genetic diversity and consequently limit long‐term survival prospects of populations (reviewed in Wright, Kalisz, & Slotte, 2013). Yet, populations of predominantly self‐fertilizing plant species like *A. thaliana* and *B. stricta* may harbor considerable levels of local quantitative and molecular genetic variation (Baron et al., 2015; Kuittinen et al., 1997; Méndez‐Vigo et al., 2013; Salmela et al., 2016; Siemens, Haugen, Matzner, & Vanasma, 2009; Song et al., 2006; Stenøien et al., 2005). Because the rate at which the population mean for a quantitative trait can change is positively correlated with the amount of genetic diversity in the trait (Falconer & Mackay, 1996; Houle, 1992), understanding the maintenance of genetic variation in the wild has become one of the key questions in modern evolutionary and conservation biology (Franks et al., 2014; Merilä & Hendry, 2014). Our results revealed not only that there was quantitative genetic variation in the high‐elevation *B. stricta* population from the Rocky Mountains but also that the magnitude of such diversity varied greatly among treatments mimicking natural growing season temperature heterogeneity. Genetic diversity in aboveground biomass was the highest under the summer temperatures of 2010, while in the treatment simulating the summer of 2009 that was on average only 0.9°C cooler than 2010, genetic variance had reduced by over 80%. For belowground biomass, we found a qualitatively similar pattern, although in this case the family effect in 2009 was a little weaker. A divergent pattern of genetic diversity emerged in the treatment simulating the warmest summer recorded at the home site of the population: Variation in both above‐ and belowground biomass was completely residual in 2012. Due to the divergence in variances, broad‐sense heritability of biomass varied between 0 and 0.31 across two treatments with a mean temperature difference of only 2.3°C.

Heritabilities and genetic variances commonly vary across environments, which complicates predicting responses to selection (Charmantier & Garant, 2005; Falconer & Mackay, 1996). Lower levels of quantitative genetic diversity may be expressed in unfavorable conditions (Charmantier & Garant, 2005; Hoffmann & Merilä, 1999). Thus, relative to the intermediate temperatures in 2010, the cooler growth environment during the month of June 2009 may have contributed to the reduced genetic variability in biomass in this year. However, the substantial biomass increase observed in response to the simulated 2012 conditions indicates that the unusually warm temperatures on their own were not detrimental to our study population. Instead, the lack of quantitative genetic diversity in biomass in this treatment may be due to the atypical high temperatures, or the novel combination of high temperature and moisture that this particular population would be unlikely to encounter in nature. Indeed, one limitation of our study is that although we programmed our chamber treatments to track closely natural temperature fluctuations that had occurred at the site, we investigated the effects of a single environmental variable only. We kept plants well‐watered throughout the experiment, but records from the weather station show that no precipitation occurred at the site in June 2012. The region experienced severe drought during the summer of 2012 (NOAA National Overview for Annual 2012), which would be expected to increase allocation to roots but also limit biomass accumulation in the shoot (Mokany, Raison, & Prokushkin, 2006; Poorter et al., 2012). Therefore, a differing response might have been observed in our experiment had we simulated natural variation in multiple environmental factors simultaneously.

In the wild, natural selection operates on total phenotypic variance shaped by both genetic and nongenetic factors (Falconer & Mackay, 1996). Because we were interested in the response of a natural population to the environmental heterogeneity of its native environment, we used wild‐collected seeds in the experiment. As a consequence, it is possible that our estimates of genetic variance also include a component due to maternal effects. Variation in the environment may result in differential maternal provisioning during seed development, the effects of which could resemble those caused by segregating genetic diversity (e.g., Bischoff & Müller‐Schärer, 2010). Further, maternal effects may be adaptive so that they enhance offspring fitness in conditions similar to those experienced by the parents (Galloway & Etterson, 2007). Drought experienced by parents has been found to enhance drought tolerance of offspring in *B. stricta* (Alsdurf, Ripley, Matzner, & Siemens, 2013), but the general importance of maternal effects to trait variation in this species is not known. While we cannot rule out potential transgenerational effects on the observed phenotypic variation, it is likely that the seeds used in the experiment matured under similar temperature and moisture conditions because all seed collections took place on the same day and within a range of a few hundred meters at a single location. Regardless of the precise underlying causes, it is noteworthy that in our experiment, the greatest potential for evolutionary responses to selection in biomass existed under intermediate temperatures and that patterns of variation under such conditions were not indicative of those in novel environmental settings caused by higher temperature.

One factor that may play a role in the maintenance of multiple phenotypically distinct lineages in a population is environmental heterogeneity on a local scale (Gillespie & Turelli, 1989; Linhart & Grant, 1996; Salmela, 2014). We hypothesized that the existence of fine‐scale genetic variation in growth in our study system could be related to among‐year variation in growing season temperature conditions in the home environment. Size is a frequent fitness proxy in plants, and shoot size has been reported to correlate moderately with reproductive effort in *B. stricta* (Siemens & Haugen, 2013); thus, changes in rank order for biomass could be expected to influence fitness ranks, too. In comparison with 2009 and 2010, we found that average aboveground biomass doubled and belowground biomass tripled under the unusually warm conditions of 2012. Biomass increase in response to warm temperatures is common in plants, especially when growing in nonlimiting conditions (Lin, Xia, & Wan, 2010; Pregitzer, King, Burton, & Brown, 2000). However, despite the significant treatment and family effects, we observed no significant family × treatment interactions in biomass across the three temperature environments. In the pairwise comparison of 2009 and 2010, the family × treatment interaction terms were significant or close to significant, but the variance components due to family were of greater magnitude than those for the interactions. This mirrors the observed treatment differences in genetic variances with little change in family rank order across temperature regimes.

The lack of family × treatment interactions in biomass suggests that temperature variation *among* growing seasons alone is unlikely to account for the genetic diversity in growth in our study system (Mitchell‐Olds, 1992). This is in accordance with a previous experiment that explored the possibility that photoperiodic and temperature variation *within* a potential growing season could maintain genetic diversity in growth traits in the same *B. stricta* population (Salmela et al., 2016). In the earlier study, month within a growing season (June, August, or September) had a large effect on overall growth, but more variation was due to family than family × treatment interactions across the three different months. In these growing season treatments, both photoperiod and temperature varied and the range of variation in temperature among treatments was more extensive than in the current study. Nevertheless, although we did not detect significant family × treatment interactions for biomass, conditional neutrality at the level of maternal family may slow the erosion of genetic variation (cf. Fry, Heinsohn, & Mackay, 1996; Schnee & Thompson, 1984). Specifically, in years similar to our 2009 and 2010 treatments, genetic variation in size would be expressed; but in years similar to 2012, genetic diversity in the population could be masked, and selection might not differentiate among distinct maternal lineages (e.g., Merilä, 1997). It is also possible that fine‐scale genetic diversity in growth in this perennial species is maintained by extensive temporal heterogeneity in low winter, spring, and autumn temperatures that could not be simulated in the chambers used, or by other abiotic or biotic factors, such as fine spatial heterogeneity in soil or competition (Baron et al., 2015; Shaw et al., 1995; Stratton, 1994).

Patterns of variation expressed for root‐to‐shoot ratio differed from the two other traits. The trait exhibited significant genetic diversity in all three treatments, and in contrast to biomass, the most genetic variation was expressed in 2012. The two other treatments had very similar levels of genetic variation, but differences in the amount of residual variation resulted in broad‐sense heritability in 2010 being almost twice as high as in 2009. The significant genetic diversity in this trait in 2012 despite the lack of such variation in its two components may be due a differing association between above‐ and belowground biomass in this treatment: The correlation between the traits was weaker and belowground biomass gain per an increase in aboveground biomass larger in 2012 than in 2010.

Similar to biomass, we detected the highest overall mean for root‐to‐shoot ratio in 2012. The trait is known to be sensitive to different environmental cues such as light and moisture, and limitations in above‐ or belowground resources often result in increased allocation to the corresponding parts of the plant (Poorter & Nagel, 2000). In our experiment, plants grew in well‐watered conditions but variable temperatures. Reduced water uptake by roots in cooler temperatures may underlie slightly increased allocation to roots in 2009 (Poorter et al., 2012). Based on among‐species patterns of variation, root‐to‐shoot ratio is expected to be negatively correlated with shoot size in herbaceous plants (Poorter et al., 2012; Wilson, 1988; see also Mokany et al., 2006); in our study focusing on a much finer level of within‐species diversity found in the wild, increases in above‐ and belowground biomass in the 2012 treatment also led to higher root‐to‐shoot ratios. Larger allocation to roots in this treatment may be related to the patterns of covariation between temperature and precipitation in the population's native environment: During a 20‐year period (1995–2014), average June temperature and precipitation increment during the month at the site were negatively correlated (*r* = −.737, *p* < .001, Figure 3B). During Junes of 2012 and 2013, the 2 years with the highest mean June temperatures, no precipitation was recorded at the site, conditions under which increased allocation to roots would be expected to be beneficial (Poorter et al., 2012). Thus, higher temperatures alone in a June environment might increase root‐to‐shoot ratios in this population.

Unlike its two components, root‐to‐shoot ratio exhibited significant family × treatment interactions across all treatments, revealing substantial genetic variation in phenotypic plasticity in this trait within the population (Falconer & Mackay, 1996). As indicated by the moderate family effect across treatments, genetic correlations were positive between treatments, but the variance component due to family × treatment interaction across all treatments was larger than that of family alone. Moreover, the interaction term was caused mainly by rank changes of families, a requirement for the maintenance of quantitative genetic diversity (Mitchell‐Olds, 1992). While our results support the idea that temperature variability can contribute to the maintenance of genetic diversity in root‐to‐shoot ratio in the *B. stricta* population, we do not have direct evidence on how selection acts on the examined traits in the wild. Increased allocation to roots may have fitness benefits at sites that experience long and cold winters (Poorter et al., 2012), especially in perennial species like *B. stricta* that have to survive over the winter in order to reproduce. Alternatively, enhanced allocation to roots could be favored under drought or low‐nutrient conditions (Lloret, Casanovas, & Peñuelas, 1999; Montesinos‐Navarro, Wig, Pico, & Tonsor, 2011; Wilson, 1988). Overall population differences in allocation observed among four high‐elevation *B. stricta* populations in southeastern Wyoming suggest the trait is linked to adaptation to cold in this region: In a chamber environment simulating late June conditions, two populations sampled close to 3,000 m had root‐to‐shoot ratios 36% higher than the other two located closer to 2,500 m, one of which was South Brush Creek (Salmela et al., 2016). This difference arose mainly from larger root biomass in the higher‐elevation populations, while shoot size did not show a similarly clear elevational grouping. Higher root‐to‐shoot ratios in populations from cooler home climates have been found in other perennial species such as *Carex aquatilis* Wahlenb. and *Picea abies* (L.) H.Karst (Chapin & Chapin, 1981; Oleksyn et al., 1998). Additional work is required on *B. stricta* to examine how biomass and its allocation are related to fitness in the spatially and temporally variable environments in the Rocky Mountains, and how environmental heterogeneity affects survival and reproductive fitness in this species. Because simulating very cold, long‐lasting and temporally variable winter conditions in growth chambers is challenging, such studies will require multiyear field experiments so that families will be exposed to their natural home site conditions.

In this experiment, our aim was to measure the potential of natural temperature heterogeneity to elicit genotype × environment interactions in a genetically diverse plant population. In conclusion, our results show that variable experimental environments affect traits differently, that the amount of genetic diversity expressed in a population can vary greatly depending on environmental conditions, that among‐trait associations may vary across environments, and that natural populations can contain significant genetic diversity in phenotypic plasticity of quantitative traits. Our study revealed limited potential of natural heterogeneity in summer temperature to change biomass ranks of maternal families in the *B. stricta* population from the Rocky Mountains. However, the magnitude of genetic variation differed across simulated years, with no genetic diversity in size in the treatment simulating the warmest summer on record at the home site of the population; such conditional neutrality of maternal families could slow the loss of genetic variation. In root‐to‐shoot ratio, family ranks within the population varied in response to natural temperature variability. Therefore, our findings support the role of genotype × environment interactions in maintaining fine‐grained quantitative genetic diversity in growth allocation. Because estimates of quantitative genetic variance are used to forecast extant populations’ adaptive responses to novel environmental conditions (Franks et al., 2014; Hoffmann & Sgrò, 2011) our observation that ecologically relevant temperature treatments affected the manifestation of genetic variation in quantitative traits is relevant to understanding the evolutionary potential within natural populations found in changing environments.
